# What Binds Cationic Photosensitizers Better: Brownian Dynamics Reveals Key Interaction Sites on Spike Proteins of SARS-CoV, MERS-CoV, and SARS-CoV-2

**DOI:** 10.3390/v13081615

**Published:** 2021-08-15

**Authors:** Vladimir Fedorov, Ekaterina Kholina, Sergei Khruschev, Ilya Kovalenko, Andrew Rubin, Marina Strakhovskaya

**Affiliations:** 1 Faculty of Biology, Lomonosov Moscow State University, 119234 Moscow, Russia; tenarra1@gmail.com (E.K.); styx@biophys.msu.ru (S.K.); ikovalenko78@gmail.com (I.K.); rubin@biophys.msu.ru (A.R.); marstr@biophys.msu.ru (M.S.); 2 Federal Scientific and Clinical Center of Specialized Types of Medical Care and Medical Technologies of the Federal Medical and Biological Agency of Russia, 115682 Moscow, Russia; 3 Institute for Persolanized Medicine, Sechenov First Moscow State Medical University (Sechenov University), 119991 Moscow, Russia; 4 Institute of Physics and Mathematics, Astrakhan State University, 414056 Astrakhan, Russia; 5 Scientific and Technological Center of Unique Instrumentation of the Russian Academy of Sciences, 117342 Moscow, Russia; 6 S.M. Nikolskii Mathematical Institute, Peoples’ Friendship University of Russia (RUDN University), 117198 Moscow, Russia

**Keywords:** SARS-CoV, SARS-CoV-2, MERS-CoV, spike protein, photosensitizer, octakis(cholinyl)zinc phthalocyanine, methylene blue, Brownian dynamics

## Abstract

We compared the electrostatic properties of the spike proteins (S-proteins) of three coronaviruses, SARS-CoV, MERS-CoV, and SARS-CoV-2, and their interactions with photosensitizers (PSs), octacationic octakis(cholinyl)zinc phthalocyanine (Zn-PcChol_8+_) and monocationic methylene blue (MB). We found a major common PS binding site at the connection of the S-protein stalk and head. The molecules of Zn-PcChol_8+_ and MB also form electrostatic encounter complexes with large area of negative electrostatic potential at the head of the S-protein of SARS-CoV-2, between fusion protein and heptad repeat 1 domain. The top of the SARS-CoV spike head demonstrates a notable area of electrostatic contacts with Zn-PcChol_8+_ and MB that corresponds to the N-terminal domain. The S-protein protomers of SARS-CoV-2 in “open” and “closed” conformations demonstrate different ability to attract PS molecules. In contrast with Zn-PcChol_8+_, MB possesses the ability to penetrate inside the pocket formed as a result of SARS-CoV-2 receptor binding domain transition into the “open” state. The existence of binding site for cationic PSs common to the S-proteins of SARS-CoV, SARS-CoV-2, and MERS-CoV creates prospects for the wide use of this type of PSs to combat the spread of coronaviruses.

## 1. Introduction

The 21st century has been followed by three outbreaks of highly pathogenic coronaviruses [1]. In 2003, the Severe Acute Respiratory Syndrome coronavirus (SARS-CoV) caused an outbreak of atypical pneumonia in 30 countries, more than 8000 people were infected, and 812 died [2]. Between April 2012 and December 2019, the Middle East Respiratory Syndrome coronavirus (MERS-CoV) infected 2499 people in 27 countries and 858 people died [3,4]. Finally, the Severe Acute Respiratory Syndrome type 2 coronavirus (SARS-CoV-2) caused the COVID-19 pandemic, which has already caused 4,265,903 deaths of 200,840,180 infected (at 9 August 2021) [5] and led to global social problems and economic losses.

Epidemic SARS-CoV, MERS-CoV, and pandemic SARS-CoV-2 are all from the *Betacoronaviruses* genus, the *Nidovirales* order, the *Coronaviridae* family. The latter includes spherical enveloped viruses with a diameter of 80 to 120 nm, the genome of which is represented with single-stranded plus-RNA ranging in size from 26.2 to 37.1 kb [6]. In SARS-CoV-2, RNA has a size of 29.9 kb, contains 14 open reading frames, and encodes 27 proteins [7]. The genomic similarity of SARS-CoV-2 with SARS-CoV and MERS-CoV is about 79% and 50%, respectively [8].

The nucleocapsid of coronaviruses is enveloped with a lipid bilayer, which originates from the host cell membranes. In beta-coronaviruses, the envelope contains three transmembrane proteins, these are envelope (E), membrane (M), and spike (S) proteins. The most abundant structural M-protein defines the shape of the viral envelope organizing CoVs assembly in the interaction with all other major structural proteins [9]. The protein E is expressed inside the infected cell and in cooperation with the M-protein is involved in viral assembly [10]. In the pathogenic human coronaviruses (hCoV), it can also mediate host immune responses [11]. Both M- and E-protein have short N-terminal ectodomains [12]. In contrast, the ectodomains of S-protein protrude from the lipid bilayer by 10–20 nm [13,14,15] and form the characteristic “crown” giving the name to this group of viruses. Direct detection of SARS-CoV-2 spike proteins in swabs from the upper respiratory tract allows rapid identification of COVID-19-positive individuals [16].

The number of spikes in SARS-CoV is 50–100 per virion; with an average spike diameter of 10 nm, the minimum distance between spikes is estimated at 14–15 nm [13]. In the prefusion state, S-protein is a homotrimer forming spikes with three heads and a trimeric stalk. Spike protomer consists of more than a thousand amino acid residues (1255 in SARS-CoV, 1273 in SARS-CoV-2, and 1353 in MERS-CoV) [6,17,18]. Each of the three spike heads is formed by S1 subunits, which bear the N-terminal domain (NTD) and the receptor binding domain (RBD). The stalk is formed by three S2 subunits, each contains a fusion peptide (FP), heptad repeats 1 and 2 (HR1 and HR2), transmembrane (TM), and cytoplasmic domains (CD) [19].

Spikes play a key role in the early stages of the replication cycle of coronaviruses, binding to host cells and fusion of membranes, which allow viruses to enter cells [20]. Thus, SARS-CoV-2 uses ACE2 as the main receptor of the host cell for binding with its S-protein [21]. ACE2 is highly expressed in lung epithelial cells and other tissues [22]. In addition to ACE2, neuropilin-1 may serve as an additional cellular mediator to promote the entry of SARS-CoV-2 [20]. Another component of the glycocalyx that serves for the primary binding of SARS-CoV-2 to the host cell is the highly negatively charged heparan sulfate, binding to which is mediated by electrostatic interactions [23]. RBD binds to heparan sulfate through a site consisting of positively charged arginine and lysine amino acid residues, adjacent to the site involved in binding to ACE2. It is assumed that SARS-CoV-2 can use negatively charged molecules of heparan sulfate for transition to an “open”, or “up” state [23]. In the “open” state, RBD is available for binding to the ACE2 receptor; this also involves electrostatic interactions [24]. Unlike SARS-CoV-2, MERS-CoV uses 5-N-acetyl-neuraminic acid to bind to cells and dipeptidyl peptidase-4 (DPP4) as the main receptor. Structurally, the corresponding binding sites are separated, and, apparently, binding to sialosides potentially increases the binding of MERS-CoV to DPP4 [25]. After binding to the receptor, the S-protein is cleaved by cellular proteases, which leads to the separation of the S1 and S2 subunits and the transition of S2 to a conformation that facilitates the process of fusion of the virus and the host cell membranes.

Since S-proteins are involved in key stages of the life cycle of coronaviruses, it is clear that host responses and a range of treatments are aimed at neutralizing their functioning, among them, anti-spike antibodies [26,27] and protease inhibitors [28] neutralizing coronavirus binding to the host cells and fusion process. Compared to SARS-CoV, in SARS-CoV-2 the S-protein region that interacts with the protease contains an additional insert with positively charged amino acid residues, and can be cleaved with a wider range of proteases, which plays a role in increasing pandemic potential of the virus [15]. Despite the fact that such an insert is located at a distance of 10–13 nm from the RBD domain [29], it also potentiates the binding of the S-protein to the negatively charged ACE-2 receptor. Thus, electrostatic interactions are involved in the most important initial stages of the replication cycle of coronaviruses—recognition, binding to host cell receptors, and priming.

Computational virology tools contribute greatly to the understanding of viral structure, infectivity and pathogenesis, and design of antiviral drugs. Computational mutagenesis revealed key mutations that affect the electrostatic properties of coronavirus spikes and RBD binding interface [30,31]. Molecular Dynamics (MD) and Monte Carlo simulations shed light on the molecular interactions of the coronavirus RBD domain with host receptors and antibodies [32], and factors that influence the fusion process [33], as well as contributed to the design of antiviral peptides [34].

Recently, we analyzed the electrostatic interactions of the photosensitizer (PS) octacationic Zn-PcChol_8+_ with the spike protein of SARS-CoV-2 using BD software ProKSim and proved PS effectiveness in the photodynamic inactivation of SARS-CoV-2 in vitro [35]. In the absence of irradiation, Zn-PcChol_8+_ exhibits the lack of antiviral activity towards avian influenza A viruses [36] as well as towards SARS-CoV-2 [35]. The fact that Zn-PcChol_8+_ itself does not influence SARS-CoV-2 infectivity coincides with the location of the main binding site of Zn-PcChol_8+_ at the junction of the head and the stalk of SARS-CoV-2 S-protein [35] separated from the sites involved in the interactions with the host cells. In contrast, a well-known antimicrobial compound methylene blue (MB), even non-photoactivated, shows in vitro activity against SARS-CoV-2 (strain IHUMI-3) at submicromolar concentrations [37]. This is apparently due to the ability of MB to inhibit interactions of the S-protein RBD with ACE2 thus blocking SARS-CoV-2 binding and entry [38]. Under irradiation, Zn-PcChol_8+_ causes the loss of SARS-CoV-2 infectivity. With a minimal studied concentration of Zn-PcChol_8+_ 1 µM and a dose of 692 nm LED light 3.75 J/cm^2^, this PS completely inactivated SARS-CoV-2 with the initial titer 5.00 lgTCID50/mL [35]. The efficiency of photodynamic inactivation of SARS-CoV-2 with Zn-PcChol_8+_ is similar to that for another enveloped virus. Indeed, Zn-PcChol_8+_ (2 µM) together with white light of 30 W halogen lamp (12 J/cm^2^) completely inactivated avian influenza A virus H5N8 with initial titer 7.125 lgTCID50/mL [36]. The second compound, MB, in the concentrations of 1.0–10.0 μg/mL (the range about 3–30 µM) with a continuous laser irradiation with wavelength λ = 662 nm (16 J/cm^2^) fully protected Vero E6 cells infected with 4 lgTCID_50_ of SARS-CoV-2 and partly protected from 5 lgTCID_50_ of SARS-CoV-2 [39]. The measured MB photodynamic activity against SARS-CoV-2 seems to be quite low taking into account that MB toxicity to SARS-CoV-2 without irradiation estimated in the same study was IC_50_ 0.22 μg/mL. Consequently, Zn-PcChol_8+_ and MB differ in antiviral action probably due to different location of their binding sites. In this study, we compare the electrostatic properties of the S-proteins (the key viral surface proteins) of three coronaviruses, SARS-CoV, MERS-CoV, and SARS-CoV-2, and their interactions with antivirals Zn-PcChol_8+_ and MB.

## 2. Materials and Methods

### 2.1. Protein 3D Models

The structural model of SARS-CoV-2 S-protein was adopted from [40]. The models of S-proteins of SARS-CoV and MERS-CoV were based on cryo-EM structures from the Protein Data Bank (PDB) with IDs 6NB3 and 5X58, respectively. The unresolved amino acid residues of the “head” domains of S-proteins were reconstructed using the i-TASSER software [41]. The “stalks” of S-proteins containing HR2, CP, and TM domains (1223–1353 for MERS-CoV and 1105–1255 for SARS-CoV) were absent in initial cryo-EM structures. We predicted their secondary structure on the basis of UniProt sequences A0A140AYW5 for MERS-CoV and P59594 for SARS-CoV using Jpred4 server [42]. Then we designed 3D structures of unresolved “stalk” parts according to the predicted secondary structure using Modeller 9.19 [43] with coiled-coil crystal structure template (PDB ID: 2WPQ) as it was previously done in [44]. The model of the Zn-PcChol_8+_ molecule was adopted from [45]. The model of the MB molecule was created using ATB web-service [46]. We used PyMOL [47] for visualization of molecular structures.

### 2.2. Brownian Dynamics Simulations

We performed rigid-body simulations of Brownian diffusion and long-range electrostatic interaction of PS molecules with coronavirus spike proteins using BD software “ProKSim” (Protein Kinetics Simulator, [48,49]). In this approach, spike protein was represented as a low dielectric area (ε = 2) with spatially fixed partial charges. Partial charges on the S-proteins were assigned in accordance with the CHARMM27 force field [50,51] using Gromacs 5.1.4. [52]. Electrostatic field of molecules was calculated using Poisson–Boltzmann formalism [53], as described in detail earlier [54]. The water solvent (dielectric constant ε = 80) with ions was described implicitly. Electrostatic cutoff radius was 3.5 nm. Ionic strength was 100 mM.

Each studied S-protein molecule was placed in a virtual reaction volume with mirror boundary conditions and dimensions of 30 × 30 × 30 nm in such a way that the entire molecule was inside the reaction volume, except its TM and CP domains. In each BD simulation, one PS molecule was initially randomly placed in the reaction volume. The BD simulation then continued until the attracting electrostatic energy reached a predetermined threshold and the resulting structure of the electrostatic encounter complex was saved for further analysis. For Zn-PcChol_8+_ the threshold was 8 kT and for MB—2 kT. Twenty thousand independent BD simulations with various initial positions of PS molecules were performed for each PS-spike protein system. Contacts between PS and particular amino acids of S-protein were characterized with a custom Python script, which identifies and counts residues of the S-protein that are within 5 A from the PS molecule throughout the obtained ensemble of structures. Contact probability for particular amino acid residue was calculated as the average fraction of contacts over the entire ensemble of structures. The contact probabilities for all amino acid residues were then visualized on the S-protein surface by color, and also on the S-protein primary sequences.

## 3. Results

### 3.1. Electrostatic Potential Fields of Coronavirus Spike Proteins and Photosensitizer Molecules

**Figure 1:**
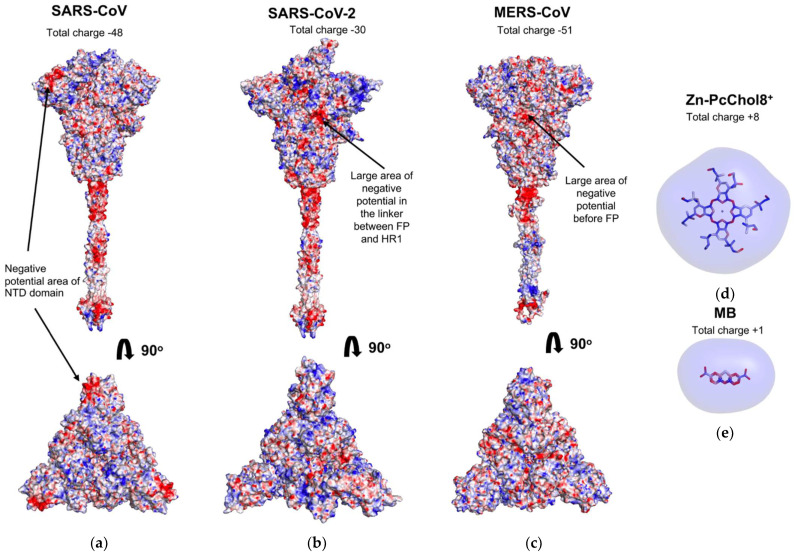
Molecular surface distribution of electrostatic potential from −100 mV (red) to +100 mV (blue) of the S-protein trimer of SARS-CoV (**a**), SARS-CoV-2 (**b**), and MERS-CoV (**c**) in the lateral view (upper panel) and top view (lower panel). The stick models of Zn-PcChol_8+_ (**d**) and MB (**e**) with equipotential electrostatic surfaces colored by red (−7 mV) and blue (+7 mV).

Figure 1 displays the surface distribution of electrostatic potential of the S-proteins of the three studied coronaviruses, SARS-CoV (A), SARS-CoV-2 (B), and MERS-CoV (C), as well as equipotential electrostatic surfaces of Zn-PcChol_8+_ (D) and MB (E) at ionic strength 100 mM. These three S-proteins have different negative charges, SARS-CoV-2 has the lowest value of −30 elementary charges compared to −48 in SARS-CoV and −50 in MERS-CoV. The total charge of the top NTD domain is −4 in SARS-CoV, +2 in SARS-CoV-2, and −3 in MERS-CoV.

S-protein stalks of SARS-CoV and SARS-CoV-2 show three large areas of negative electrostatic potential, with the largest negatively charged area located at the stalk and head connection, whereas the MERS-CoV S-protein stalk demonstrates only two major areas of negative potential, one at the stalk and head connection and the other at the cytoplasmic domain. The electric charge is highly heterogeneously distributed on the surface of the S-protein head. Nevertheless, areas with a pronounced negative potential are observed on the head of each of the considered coronaviruses. The noteworthy negatively charged area on the NTD-related surface of the SARS-CoV S-protein head is generated by aspartate and glutamate residues Asp15, Asp17, Asp23, Asp24, Asp134, Asp154, Asp243, and Glu131. There are no similar negatively charged regions on the NTD of SARS-CoV-2 and MERS-CoV, which is explained by the absence of some corresponding negatively charged amino acid residues. Instead, the head of MERS-CoV S-protein has a large area of negative electrostatic potential at the linker subdomain between RBD and FP (amino acid residues 676–686, 708–723, and 879–942). SARS-CoV-2 S-protein has the similarly located negatively charged area constituted by amino acid residues of linker between FP and HR1 domains.

The PS molecules MB and Zn-PcChol_8+_ demonstrate only positive electrostatic potential surface, with +1 at MB and +8 total charge generated by choline substitutes in Zn-PcChol_8+_.

### 3.2. Binding of Photosensitizer Molecules to Coronavirus S-Proteins

**Figure 2:**
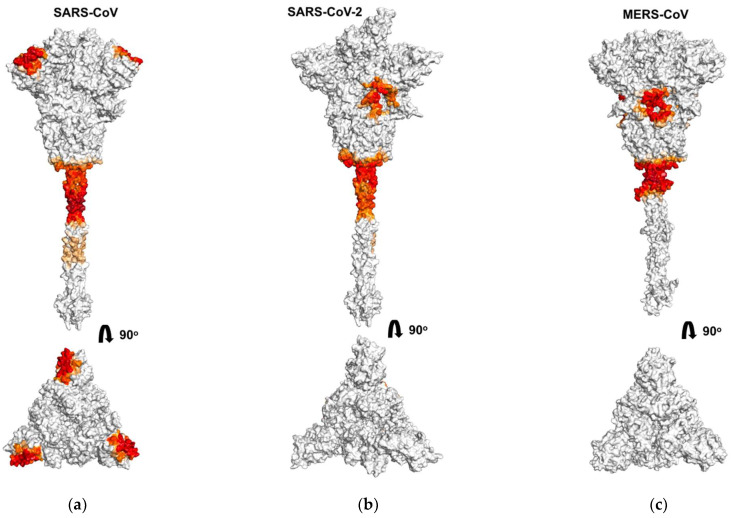
Molecular surfaces of coronavirus spike proteins of SARS-CoV (**a**), SARS-CoV-2 (**b**), and MERS-CoV (**c**) with depicted areas of Zn-PcChol_8+_ binding in the lateral view (upper panels) and in the top view (lower panels). The regions of PS interactions with S-protein surface are colored by gradient from orange to red in dependence of contact probability.

**Figure 3:**
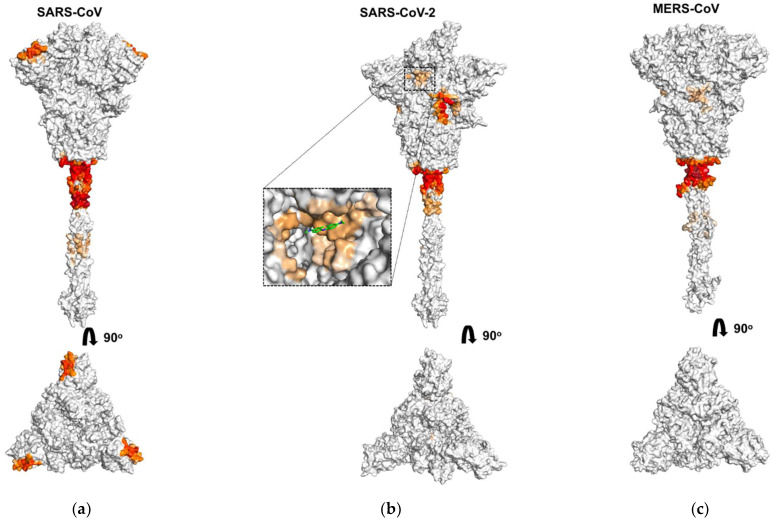
Molecular surfaces of coronavirus spike proteins of SARS-CoV (**a**), SARS-CoV-2 (**b**), and MERS-CoV (**c**) with depicted areas of MB binding in the lateral view (upper panels) and in the top view (lower panels). The regions of PS interactions with S-proteins surfaces are colored by gradient from orange to red in dependence of contact probability. The inset shows one possible energetically favorable position of MB molecule (colored in green) in the binding cavity.

**Figure 4:**
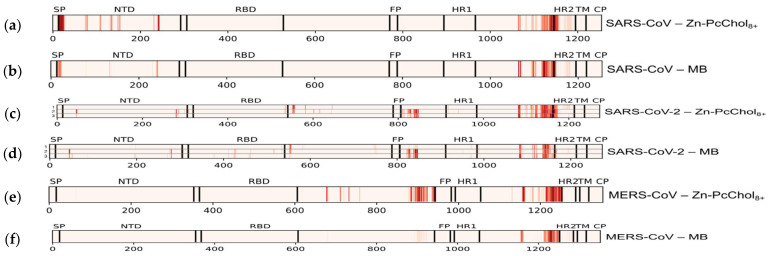
Amino acid residues in primary sequences of SARS-CoV (**a**,**b**), SARS-CoV-2 (**c**,**d**), and MERS-CoV (**e**,**f**) with assignment of functional domains colored depending on the probability of PS binding. (c1,d1) are related to the SARS-CoV-2 S-protein protomers in the “open” state, and (c2,c3,d2,d3)—to the S-protein protomers in the “closed” state.

We analyzed the binding of octacationic and monocationic PSs to the S-proteins of three coronaviruses, SARS-CoV, SARS-CoV-2, and MERS-CoV. Figure 2, Figure 3 and Figure 4 show amino acid residues involved in contacts with PSs on the surface of S-proteins (Figure 2 and Figure 3) and in primary sequences with a domain structure (Figure 4), colored depending on the probability of PSs binding. For the SARS-CoV-2 S-protein, amino acid residues involved in contacts with PSs are given for each of the three spike protomers, one of them in the “open” state (Figure 4(c1,d1)) and two others in the “closed” state (Figure 4(c2,c3,d2,d3)).

Figure 2 shows the areas on the S-protein surface with which Zn-PcChol_8+_ molecules form encounter complexes with an electrostatic attraction energy exceeding 8 kT. As MB possesses lower total electric charge than Zn-PcChol_8+_, it has lower values of electrostatic energy when interacting with S-proteins. Therefore, we were able to detect encounter complexes of MB with S-proteins only with a lower threshold (2 kT) of electrostatic attraction energy. In general, the binding sites of this PS resemble those for Zn-PcChol_8+_, although there are some differences. The most prominent attractive areas at the junction of the stalk and the head of S-proteins are present both for Zn-PcChol_8+_ and MB.

As previously found for the interaction of Zn-PcChol_8+_ with SARS-CoV-2 [35], the molecules of this PS formed electrostatic encounter complexes with certain areas of the S-protein surface in two other investigated coronaviruses. The only one common binding area for S-protein of all three coronaviruses is the vast area of negative electrostatic potential at the junction of the stem and the head. This area can also be clearly seen in Figure 4 at the linker between HR1 and HR2 domains for all three S-proteins. Note that there is almost no difference in PS binding to this area for the protomers in the “open” and the “closed” states.

Similar to the binding of Zn-PcChol_8+_ to SARS-CoV-2 S-protein [35], the molecules of this PS formed electrostatic encounter complexes with the largest area of negative electrostatic potential at the head of the S-protein of MERS-CoV (Figure 2c, upper panel) just before the FP domain (Figure 4e). Unlike SARS-CoV-2 and MERS-CoV, this negatively charged region is absent on the S-protein of SARS-CoV. However, the top of the SARS-CoV spike head demonstrates a notable area of electrostatic contacts with Zn-PcChol_8+_ (Figure 2a) and MB (Figure 3a). This area corresponds to the NTD (Figure 4a,b).

**Figure 5:**
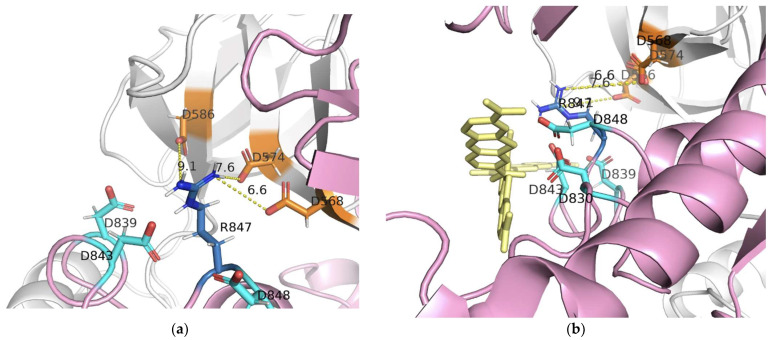

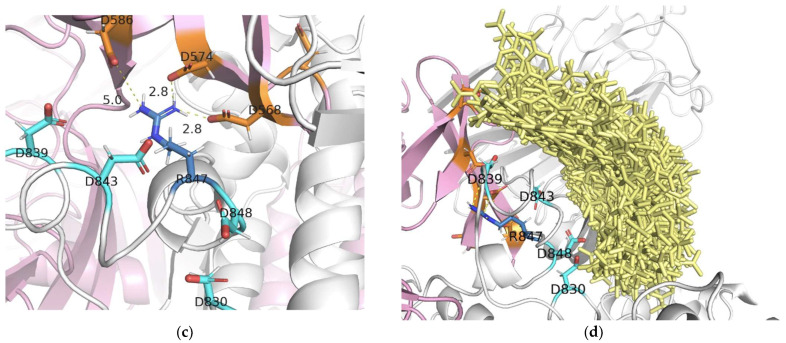
Arg847 (shown in blue) of the S-protein protomer of SARS-CoV-2 in the “open” (**a**,**b**) and the “closed” (**c**,**d**) states and its environment. Aspartate residues belonging to the same S-protein protomer as Arg847 are colored in cyan, aspartate residues belonging to the adjacent S-protein protomer are colored in orange. The protomer of S-protein in the “open” state is colored in pink, the adjacent protomer in the “closed” state in white. Plates (**b**,**d**) show possible positions of MB molecules relative to the spike protein. MB molecules are rendered by sticks and colored in yellow. The dashed yellow lines show distances between some contacting amino acid residues.

The largest area of negative electrostatic potential at the head of the S-protein of SARS-CoV-2 also attracts MB molecules (Figure 3b) as it was for Zn-PcChol_8+_ [35]. The attractive ability of the similar area of MERS-CoV S-protein for MB is almost totally reduced (Figure 3c). The S-protein protomers of SARS-CoV-2 in “open” and “closed” conformations demonstrate a different ability to attract PS molecules (Figure 4c,d). The mentioned above largest area of negative electrostatic potential at the head of the S-protein protomers in the “closed” conformation attracts Zn-PcChol_8+_ (Figure 4(c2,c3)) and MB (Figure 4(d2,d3)). This area is located just after the FP domain. The S-protein protomer in the “open” conformation (Figure 4(c1,d1)) hardly attracts PS molecules in this region. To understand the nature of this difference, we analyzed the spatial conformation of distinct amino acid residues generating electrostatic potential in this area for both “open” and “closed” states. Arg847 of the S-protein protomer in the “open” state is exposed to solution and shields negatively charged residues Asp830, Asp839, Asp843, Asp848 of the same protomer, therefore, positively charged PS molecules hardly form electrostatically favorable complexes with this area (Figure 4(c1,d1) and Figure 5a,b). In the “closed” state, Arg847 forms hydrogen bonds with D574, D568, and D586 of the adjacent S-protein protomer. Thus, Arg847 is not exposed into solution and does not impose such a serious shielding effect on the negative electrostatic field of this area (Figure 5c,d) and PS binding (Figure 4(c2,c3,d2,d3)).

In contrast with Zn-PcChol_8+_, MB possesses the ability to penetrate inside the pocket formed as a result of SARS-CoV-2 RBD domain transition into the “open” state (Figure 3b). This MB binding site possesses a complex labyrinthine geometry that is composed by amino acid residues of NTD, RBD, and HR1 domains belonging to protomers in the “open” and “closed” states (Figure 4d).

## 4. Discussion

In our study, we used BD to simulate the binding of water-soluble cationic PSs to coronavirus S-proteins that are viral structures mostly protruded into a water environment. The S-proteins of all three studied coronaviruses have a significant negative total charge formed as a result of the excess of the number of negatively charged amino acid residues (Asp + Glu) over positively charged ones (Arg + Lys). This electric charge is highly heterogeneously distributed on the S-protein surface and there are only a few areas of pronounced negative electrostatic potential (colored in red in Figure 1). The largest of them, common to all three coronaviruses, is located at the connection of the S-protein stalk and the head on the linker between the HR1 and HR2 domains, adjacent to HR2. Consequently, it attracts the majority of Zn-PcChol_8+_ and MB molecules and constitutes the main binding site for both cationic dyes located at a distance of about 10 nm from the viral membrane. Zn-PcChol_8+_ and MB are both the Type II PSs, which produce singlet oxygen under red light irradiation. Earlier we proposed [35] that the location of PS in this binding site promotes effective oxidative damage to both the S-protein and the viral membrane. In vitro studies confirmed Zn-PcChol_8+_ to be highly effective in SARS-CoV-2 photodynamic inactivation [35].

The photodynamic activity of MB towards MERS-CoV [55] and SARS-CoV-2 [39] is a well-known experimental fact. However, as it has been shown recently for SARS-CoV-2, MB antiviral activity occurs even in the absence of light [37,39]. Unlike MB, Zn-PcChol_8+_ in the concentrations up to 5 µM has no antiviral activity without irradiation [35]. The mechanism of the “dark” MB antiviral effect on the infectivity of SARS-CoV-2 is elusive. One of the possible explanations comes from our simulations. We found that MB molecules are able to bind to the internal cavity inside the SARS-CoV-2 S-protein head (the inset in Figure 3), which opens for binding as the result of RBD transition into the “open” conformation. The binding of MB to the RBD domain may affect interaction of the S-protein with the ACE2 receptors on the host cell, thus, reducing virus infectivity. In contrast to MB, the much larger Zn-PcChol_8+_ molecules did not appear in this cavity in our simulations. This agrees with our finding that Zn-PcChol_8+_ lacks dark antiviral activity.

We also analyzed the binding of positively charged dyes to other S-protein key sites, such as the cleavage site and FP. It turned out that in all studied S-proteins in the loci between RBD and FP, Zn-PcChol_8+_ and MB do not bind directly to the cleavage sites, apparently due to the presence of positively charged arginine residues in these sites. However, we cannot completely exclude the effect of cationic dyes on S-protein cleavage in SARS-CoV-2 and MERS-CoV, since one can see several binding sites between RBD and FP (Figure 4), although not directly at the cleavage sites. In SARS-CoV, subdomains between RBD and FP do not bind Zn-PcChol_8+_ and MB, so, it is unlikely that cationic dyes would affect S-protein cleavage in this coronavirus. The facts that Zn-PcChol_8+_ and MB bind to the region just after FP in SARS-CoV-2 and that the notable Zn-PcChol_8+_ binding site in MERS-CoV S-protein appears just before FP, as well as the localization of the main binding sites adjacent to HR2 (Figure 4) mean that cationic dyes can potentially affect the process of fusion, but this hypothesis needs experimental proof.

The ability to recognize a variety of host cell receptors greatly impacts virus infectivity. Along with RBD binding to main protein receptors, NTDs of MERS-CoV can accommodate sialosides at neutral pH [56], while NTD of SARS-CoV uses only ACE2 receptor to bind to host cells [57]. In coronaviruses, NTDs of S-proteins have β-sandwich structure modified by various variable loops that affect recognition and binding to the host cell [25]. NTDs of SARS-CoV and SARS-CoV-2 show much lower (53.5%) homology compared to full-length S-proteins [58]. The total charge of the NTDs also varies among the studied coronaviruses, from −4 in SARS-CoV to +2 in SARS-CoV-2. According to the aligned NTD sequences, the key negatively charged residues that make up the prominent region on the head of the SARS-CoV S-protein in both SARS-CoV-2 and MERS-CoV are largely replaced by neutral amino acid residues. The substitution of the negative Asp23 in SARS-CoV with Arg21 or Lys27 in SARS-CoV-2 and MERS-CoV, respectively, is especially intriguing. The heterogeneous distribution of positively and negatively charged amino acid residues in the NTDs of SARS-CoV-2 and MERS-CoV results in the lack of the noticeable areas of negative electrostatic potential. Both Zn-PcChol_8+_ and MB were rare guests in the SARS-CoV-2 NTD and did not bind to MERS-CoV NTD. In contrast, in each of the NTDs of SARS-CoV S-protein trimer, negatively charged amino acid residues form condensed areas of negative potential, attracting Zn-PcChol_8+_ and MB molecules (Figure 2a and Figure 3a). At the same time, the presence of such negatively charged regions can prevent SARS-CoV NTD contacts with negatively charged cellular receptors. This is in line with the experimental fact that NTD of SARS-CoV does not bind to sialic acid [57,58].

Finally, we found several PS binding sites in different S-protein domains involved in the initial processes of viral replication cycle—the primary recognition of cell receptors, binding to the main receptor, cleavage, and membrane fusion. The domain structure determines the selective binding of dye molecules and, thus, the nearby targets that can be damaged with singlet oxygen when illuminating the bound PS. In all three studied S-proteins, for both cationic dyes with photodynamic activity, Zn-PcChol_8+_ and MB, we found a major binding site at the connection of the S-protein stalk and the head adjacent to the HR2 domain. The existence of such main binding site for cationic PSs, common to the S-proteins of SARS-CoV, SARS-CoV-2, and MERS-CoV, creates prospects for the wide use of PSs as photodesinfectants to combat the spread of coronaviruses. Our results can be useful in studying the initial electrostatic interactions of other cationic antiseptics with surface proteins of coronaviruses [59]. Brownian dynamics can help in studying the interaction of potential cationic antiviral drugs with negatively charged receptors of host cells that inhibit viral binding. A rational choice between the affinity for the receptors of host cells and viral structures can significantly increase the effectiveness of antiviral drugs, the mechanism of action of which involves electrostatic interactions.

## Data Availability

Data available in a publicly accessible repository.
